# Risk Factors of Incidental Cholangiocarcinoma in Primary Sclerosing Cholangitis: A Cross-Sectional Study with ROC Curve Analysis

**DOI:** 10.34172/aim.34922

**Published:** 2026-01-01

**Authors:** Fardad Ejtehadi, Saeid Hashemieh, Ali Reza Safarpour, Sara Shojaei-Zarghani, Salar Azadnik, Alireza Shamsaeefar, Nima Rahimikashkooli

**Affiliations:** ^1^Gastroenterohepatology Research Center, Shiraz University of Medical Sciences, Shiraz, Iran; ^2^Colorectal Research Center, Shiraz University of Medical Sciences, Shiraz, Iran; ^3^Internal Medicine Department, Shiraz University of Medical Sciences, Shiraz, Iran; ^4^Transplant Research Center, Shiraz University of Medical Sciences, Shiraz, Iran

**Keywords:** CA-19-9 antigen, Cholangitis, Cholangiocarcinoma, Sclerosing

## Abstract

**Background:**

Cholangiocarcinoma (CCA) is the most common malignancy in patients with primary sclerosing cholangitis (PSC). It is typically associated with low survival rates due to late diagnosis. This study aimed to evaluate the predictors of incidental CCA in PSC patients.

**Methods:**

In this cross-sectional study, we included 425 patients aged 18 years or older who underwent liver transplant with a confirmed diagnosis of PSC. Demographic data, pre-transplant clinical features, and para-clinical evidence were obtained from medical records. Pathology experts examined livers removed during transplantation, and CCA was diagnosed accordingly. Multivariable logistic regression and receiver operating characteristic (ROC) curve analyses were conducted to assess the risk factors for incidental CCA and the effectiveness of carbohydrate antigen 19-9 (CA 19‒9) in predicting CCA, respectively.

**Results:**

Of the 425 included patients, 29 had PSC-CCA and 396 patients had PSC alone. According to the multivariable logistic model, CA 19‒9 (odds ratio [OR]=1.001, 95% confidence interval [CI]: 1.000‒1.001, *P*-value=0.041) and weight loss (OR=4.712, 95% CI: 1.392 to 15.947, *P*-value=0.013) were significantly associated with CCA development. ROC curve analysis also revealed that CA 19‒9 could predict CCA (AUC=0.737; 95% CI: 0.689‒0.782) at an optimal cut-off point above 46 U/mL, with a good sensitivity (68%, bootstrapped 95% CI: 62‒74%) and specificity (72.32%, bootstrapped 95% CI: 68‒76%).

**Conclusion:**

We found that CA 19‒9 and weight loss are independent predictors of incidental CCA in PSC patients, and a CA 19‒9 level above 46 U/mL has relatively good predictive power for incidental CCA.

## Introduction

 Primary Sclerosing Cholangitis (PSC) is a chronic inflammatory disease involving the intrahepatic and/or extrahepatic bile ducts. This can lead to bile stasis, strictures, fibrosis, and cirrhosis of the liver, often ultimately requiring liver transplantation (LT).^1^ In the general population, the incidence rate and prevalence of PSC is reported to be 0.75 and 11.16 per 100,000 persons, respectively.^2^ PSC is typically diagnosed in individuals between the ages of 30 and 40 years.^3^ The etiology of PSC is still unknown and there is no current consensus about it. However, genetic, epigenetic, environmental, and immunological factors seem to be major contributors.^4,5^ At the present time, there is no proven effective treatment for PSC and the major management strategies of the disease are based on controlling the symptoms and complications.

 Peribiliary fibrosis in PSC can result in strictures of the biliary tree and accumulation of bile acids, leading to DNA damage in cholangiocytes and development of cholangiocarcinoma (CCA). CCA often occurs in individuals with genetic and epigenetic predispositions, as well as chronic inflammation of the liver (viral hepatitis, cirrhosis) or bile duct (PSC, hepatolithiasis).^6^ It is the most common hepatobiliary malignancy and a leading cause of death in PSC patients.^7,8^ The annual occurrence of CCA in PSC patients varies from 0.5 to 1.5 per 100 individuals, which is significantly higher, by 10 to 1000 times, than the incidence observed in the general population.^9,10^ Previous studies conducted mainly in European countries and North America have suggested that large-duct abnormalities, age, male sex, concurrent ulcerative colitis, alcohol and tobacco use, as well as hepatitis B or C infection are well-known risk factors for PSC-associated CCA.^10^ However, due to the geographic or ethnic differences, further studies should be conducted in Asia to better understand other risk factors.

 Currently available screening tools for CCA in PSC patients include serological testing, imaging techniques, and evaluation of patient symptoms. However, these methods have low specificity and sensitivity, leading to misdiagnosis or delayed detection in a significant number of patients.^11^ Due to the detrimental effects of incidentally found tumors in liver explants on postoperative tumor recurrence and survival, identifying high-risk PSC patients is necessary.^12^ Therefore, the aim of this study was to identify and evaluate biomarkers that can predict incidentally-found CCA in PSC patients before LT and provide prognostic information.

## Materials and Methods

 In this retrospective analytic cross-sectional study, we included patients aged 18 years or older who underwent liver transplant with a confirmed diagnosis of PSC by MRCP^13^ who were admitted to Abu-Ali Sina and Namazi Hospitals in Shiraz, Iran between 2015 and 2020 and had available medical records. Incidental CCA refers to cancers discovered in the liver explants post-liver transplantation without prior awareness of their existence.^12^ Patients with a prior CCA diagnosis before LT, those without a PSC diagnosis based on histological criteria, and patients with secondary causes of PSC, any history of malignancy, and evidence of active infection or chronic liver disease that prevented LT were excluded from the study. The study was approved by the ethics committee of Shiraz University of Medical Sciences, Shiraz, Iran (Code: IR.SUMS.MED.REC.1400.442).

 The sample size was calculated using the following formula:


$$n=\frac{Z^{2}P\left(1-P\right)}{d^{2}}$$

Given the reported prevalence of CCA in Iran (8.8%) ^14^, 95% confidence interval (CI), and precision (d) of 0.027, the sample size was estimated to be 393. With consideration of a dropout, 425 subjects were included in the study.

 Demographic information and detailed pre-transplant clinical features, symptoms, and para-clinical evidence were extracted from the electronic medical records. Individual telephone calls were also made to obtain additional information. After explaining the importance of the research and obtaining verbal informed consent, we completed our available data. Standard methods were used for measuring laboratory parameters including, liver function tests, total and direct bilirubin, albumin level, complete blood count, and Carbohydrate Antigen 19‒9 (CA 19‒9). The patient were monitored with ultrasonography every three months and underwent MRCP every 6-12 months, or sooner if there were any persistent changes in alkaline phosphatase or CA 19‒9 level. The Mayo and Model for End-Stage Liver Disease (MELD) scores were also calculated to better understand disease prognosis.^15^ To further evaluate PSC and potentially detect CCA, explanted livers were sent for pathological examination. The histopathological reports were obtained from expert liver pathologists, and patients were categorized into two groups of with or without CCA, accordingly. Weight loss was defined as an unintended decrease of more than 10 pounds in body weight over the past 12 months. Body weight was recorded prior to breakfast in the morning.

**Figure 1:**
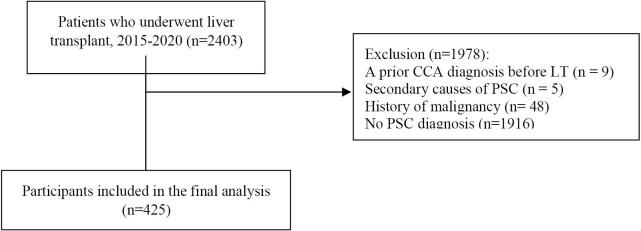
Flow diagram of study selection process

 We used IBM SPSS (version 25.0) and R (version 4.3.1) to conduct statistical analysis. The normality of data was assessed using Kolmogorov–Smirnov test. Non-parametric quantitative and qualitative data are presented as median (interquartile range) and number (percentages), respectively. In order to examine whether there were differences in data between subjects with and without CCA, we used Mann-Whitney U test or Chi-square test depending on the nature of the variable. Assumptions for logistic regression were verified, including linearity in the logit for continuous predictors (including: CA 19‒9) using the Box-Tidwell test (*P* > 0.05 indicating no violation), multicollinearity assessed via variance inflation factors (VIF = 1.12 for CA 19‒9 and weight loss), and influential outliers evaluated using Cook’s distance (no values > 1). Variables that differed between individuals with and without CCA, or those selected based on the literature, were included in the multivariable logistic regression model. Backward stepwise selection was employed (*P*-values for entry and removal set to 0.10 and 0.05, respectively), with results cross-verified using forward selection and Akaike Information Criterion (AIC) for model stability. To address potential rare events bias due to the low prevalence of CCA (~6.8%), Firth’s penalized likelihood logistic regression was employed. The results are reported as odds ratios (ORs) with a 95% CI. Model fit was evaluated using the Hosmer-Lemeshow goodness-of-fit test (*P* > 0.05 indicating good fit) and Nagelkerke pseudo-R^2^. *Post-hoc* power calculations for key predictors were performed to assess statistical power. We also utilized a Receiver Operating Characteristic (ROC) curve to assess the effectiveness of CA 19‒9 in predicting CCA. The predictive power was evaluated through the Area Under the Curve (AUC), with bootstrapped 95% CIs (1,000 replications) for AUC, sensitivity, and specificity. The clinically relevant cut-off point for predicting CCA was determined by maximizing sensitivity + specificity (Youden index). Additional metrics, including positive predictive value (PPV), negative predictive value (NPV), positive likelihood ratio (LR+), and negative likelihood ratio (LR-), were calculated at the optimal cutoff. Sensitivity analyses were conducted, including exclusion of influential outliers (based on Cook’s distance) to confirm robustness. *P*-value < 0.05 was considered significant. The study adheres to the Strengthening the Reporting of Observational Studies in Epidemiology (STROBE) guidelines for cross-sectional studies, with the flow diagram of participant inclusion provided in Figure 1.

## Results

 In the present study, 425 subjects (29 patients with and 396 patients without CCA) were included. As is reported in Table 1, sex distribution, age, duration of PSC, the occurrence of repeated cholangitis, biochemical and hematological parameters, and MELD and Mayo scores were similar between the two groups.

**Table 1:** Patients’ demographic and clinical characteristics

**Variable**	**With CCA (** ***n*** **=29)**	**Without CCA (** ***n*** **=396)**	***P*** **-value**
Sex (Male), *n *(%)	21 (72.4)	234 (59.1)	0.157 ^a^
Weight loss, *n* (%)	24 (82.80)	230 (58.10)	**0.009 ** ^a^
Repeated cholangitis, n (%)	17 (58.60)	231 (58.30)	0.976 ^a^
Age (year), median (IQR)	39.00 (33.00‒53.00)	38.00 (31.00‒47.00)	0.216 ^b^
Duration of PSC (year), median (IQR)	2.00 (2.00‒5.00)	4.00 (2.00‒6.00)	0.206^b^
Hb (g/dL), median (IQR)	12.00 (11.00‒13.00)	11.00 (10.00‒13.00)	0.059^b^
WBC (10^9^/L), median (IQR)	7100.00 (5425.00‒9800.00)	6250.00 (4500.00‒8485.00)	0.132^b^
INR, median (IQR)	1.00 (1.00‒1.00)	1.00 (1.00‒2.00)	0.177^b^
Platelets (10^9^/L), median (IQR)	29000.00 (146000.00‒256000.00)	162000.00 (100000.00‒257000.00)	0.113^b^
Albumin (g/dL), median (IQR)	4.00 (3.00‒4.00)	3.00 (3.00‒4.00)	0.061^b^
ALT (U/L), median (IQR)	60.00 (36.50‒90.50)	62.00 (33.00‒97.00)	0.846^b^
AST (U/L), median (IQR)	71.00 (46.00‒120.50)	83.00 (54.00‒125.00)	0.362^b^
ALK (U/L), median (IQR)	976.00 (483.00‒1252.50)	706.50 (423.25‒1150.00)	0.300^b^
Total bilirubin (mg/dL), median (IQR)	3.00 (1.00‒17.50)	5.00 (2.00‒13.00)	0.206^b^
Direct bilirubin (mg/dL), median (IQR)	1.00 (1.00‒11.00)	3.00 (1.00‒8.00)	0.337^b^
MELD score, median (IQR)	16.00 (11.00‒21.00)	18.00 (13.00‒22.00)	0.314^b^
CA19‒9 (U/mL), median (IQR)	89.00 (18.00‒399.50)	19.50 (5.00‒60.00)	**<0.001** ^b^
Mayo Score, median (IQR)	1.00 (0.50‒3.00)	2.00 (1.00‒3.00)	0.131^b^

ALT, Alanine transaminase; AST, Aspartate aminotransferase; ALK, Alkaline phosphatase; CA 19-9, Carbohydrate antigen 19-9; CCA, cholangiocarcinoma; Hb, Hemoglobin; INR, International normalized ratio; IQR, Interquartile range; MELD, Model for end-stage liver disease; PSC, Primary Sclerosing Cholangitis. Between-group analyses of qualitative and quantitative data were conducted using ^(a)^ Chi-square and ^(b)^ Mann-Whitney U test, respectively. *P*-value < 0.05 was considered significant.

 Nevertheless, a higher proportion of PSC-CCA patients had experienced weight loss compared to PSC patients (82.80% of PSC-CCA patients versus 58.10% of PSC, *P*-value = 0.009). Furthermore, CA 19‒9 levels were significantly higher in the PSC-CCA group compared to the control (*P*-value < 0.001).

 In the multivariable model, two independent risk factors were identified to be significantly associated with CCA development, including CA 19‒9 (OR = 1.001 per unit [95% CI: 1.000‒1.001],* P*-value 2 = 0.041, corresponding to OR = 1.010 per 10 units [95% CI: 1.000‒1.020] and OR = 1.105 per 100 units [95% CI: 1.000‒1.221]) and weight loss (OR = 4.712, 95% CI: 1.392 to 15.947, *P*-value = 0.013) (Table 2). The 95% CI for weight loss indicates a significant association but with imprecision due to the low event rate. The model demonstrated good fit (Hosmer-Lemeshow test: χ^2^ = 5.89, *P* = 0.66) with a Nagelkerke pseudo-R^2^ = 0.18

**Table 2:** Multivariable logistic regression for risk factors of CCA diagnosis

**Variables**	**OR (95% CI)**	***P*** **-Value**
CA 19‒9 (continuous)	1.001 (1.000 to 1.001)	0.041
Weight loss (no *vs.* yes)	4.712 (1.392 to 15.947)	0.013

CA 19‒9, Carbohydrate antigen19‒9; CCA, Cholangiocarcinoma; CI, Confidence Interval; MELD, Model for end-stage liver disease; OR; Odds Ratio. Age, sex, hemoglobin, CA 19-9, weight loss, MELD score, and albumin were included in the logistic regression. Only the above variables were retained in the final model. *P*-value < 0.05 was considered significant.

**Figure 2:**
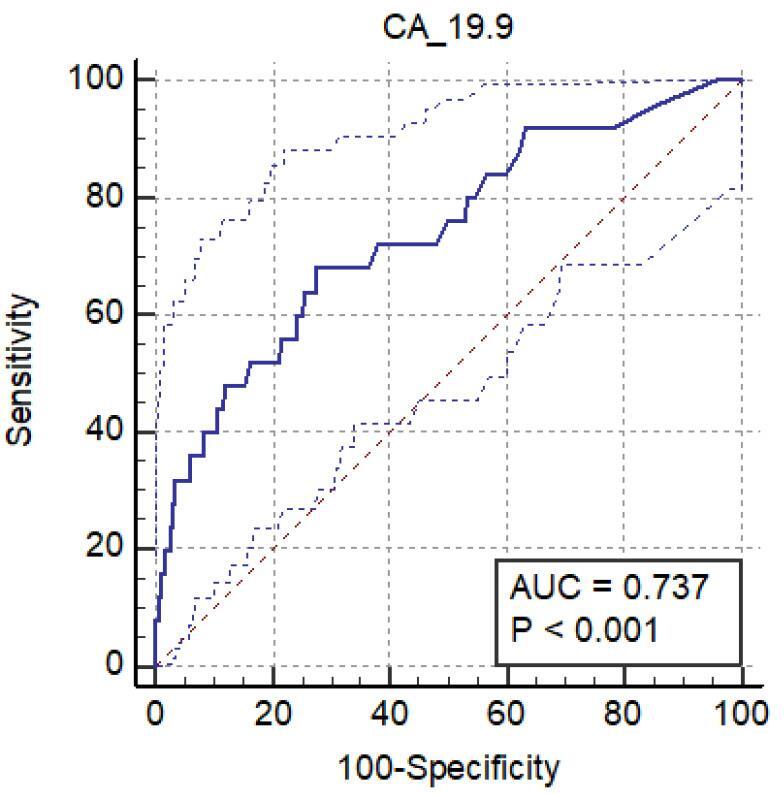
ROC showing the predictive performance of CA 19-9 for CCA. AUC; Area Under the Curve; CA 19‒9; Carbohydrate antigen 19-9; CCA, Cholangiocarcinoma; ROC, Receiver Operating Characteristic

 We also assessed the performance of CA 19‒9 in predicting CCA, as indicated in Figure 2 and Table 3. We found that CA 19‒9 could predict CCA (AUC = 0.737; 95% CI: 0.689‒0.782) at an optimal cut-off point above 46 U/mL, with good sensitivity (68%, bootstrapped 95% CI: 62-74%) and specificity (72.32%, bootstrapped 95% CI: 68‒76%), with a Youden Index of 40.3%. At the > 46 U/mL cutoff, PPV was 15.4%, NPV was 96.8%, positive likelihood ratio was 2.45 and negative likelihood ratio was 0.43, highlighting its utility in ruling out CCA.

**Table 3:** Predictive performance of CA 19-9 for CCA

Area under the ROC curve (AUC)	0.737
Significance level P (Area = 0.5)	< 0.001
95% Confidence interval	0.689‒0.782
Standard error	0.0563
Youden index J	0.4032
Associated criterion	> 46
Sensitivity	68.00
Specificity	72.32

AUC, Area Under the Curve; CA 19‒9, Carbohydrate antigen 19‒9; CCA, Cholangiocarcinoma; ROC, Receiver Operating Characteristic. *P*-value < 0.05 was considered significant.

 In a separate analysis, we classified CA 19‒9 levels as either ≤ 46 U/mL or > 46 U/mL, and incorporated this variable into a multivariable model. The findings indicated that a CA 19‒9 level above 46 was significantly associated with 5.244-fold increased odds of CCA (95% CI: 2.167‒12.690, *P*-value < 0.001).

## Discussion

 PSC is recognized as a disease with the potential to progress to malignancy.^16^ CCA is the most prevalent type of cancer in individuals with PSC and constitutes a major cause of mortality in these patients.^17,18^ Despite the availability of multiple diagnostic techniques, early identification of CCA in PSC patients and prioritizing those at lower risk of developing CCA remain significant clinical challenges.^19^ This study aimed to evaluate the importance of various factors in predicting incidental CCA in PSC patients.

 We found that 6.8% of the PSC patients had incidental CCA at LT, which was almost similar to the previous literature.^12,20,21^ Clinicians commonly suspect CCA in PSC patients who exhibit rapidly deteriorating hepatic function, characterized by worsening jaundice, loss of body weight, and abdominal discomfort.^10^ While CA 19‒9 and weight loss emerged as independent predictors in this cross-sectional study, causal inferences are limited and these should be viewed as associations requiring prospective confirmation. The significance of CA 19‒9 (*P* = 0.040) was borderline and may not survive strict multiplicity; however, given the exploratory nature and biological plausibility, it warrants further investigation. In contrast to our results, Charatcharoenwitthaya *et al*. indicated that presence of weight loss was not significantly different between PSC patients with and without early-stage CCA.^22^ Furthermore, these symptoms are non-specific and may also be observed in benign PSC or gynecological cancers.^23^ Therefore, additional studies should be conducted to identify more sensitive and specific markers for accurate CCA diagnosis.

 In the current study, CA 19‒9 showed moderate predictive power (AUC = 0.737) but it should not be used in isolation for diagnosis, as the Youden index (0.403) indicates room for improvement. CA 19‒9 is a cell surface glycoprotein complex synthesized by pancreatic ductal cells, biliary system, and epithelial cells located in the stomach, colon, uterus, and salivary glands. It is widely evaluated as a tumor marker for diagnosing CCA.^24^ Our results align with a previous meta-analysis study involving 31 individual studies, which concluded that CA 19‒9 could serve as a non-invasive tool for predicting CCA.^25^ According to that study, CA19‒9 had sensitivity and specificity ranging from 33% to 100% and from 31% to 100%, respectively, across studies. The pooled results showed a sensitivity of 0.72 (95% CI: 0.70–0.75) and specificity of 0.84 (95% CI: 0.82–0.85). The sensitivity of CA 19‒9 varied across different subgroups: 62% in Europeans, 71% in Americans, and 74% in Asians. The cutoff value for CA 19‒9 also varied from 20 to 200 U/mL. These differences could be due to variations in geographic locations, demographic characteristics, and health status of control groups. It should be noted that the Youden Index (sensitivity + specificity – 1) fell below 50% at our reported cut-off point, indicating a low balance between sensitivity and specificity. As a result, CA 19‒9 > 46 cannot be deemed a diagnostic criterion, but also recommended to use this biomarker alongside other relevant tests. In this regard, CA 19‒9 is noted to be influenced by other diseases affecting bilirubin levels, which may limit its diagnostic value.^26^

 In the present study, we observed no significant association between various factors investigated (such as age, sex, duration of PSC, and liver function tests) and the risk of CCA. These findings are consistent with a previous cohort study conducted by Burak *et al*., which followed PSC patients for a median duration of 11.5 years until the diagnosis of CCA or LT.^27^ However, some studies have reported age, male sex, longer duration of PSC, higher levels of total and direct bilirubin, and higher Mayo score as independent predictors for CCA development in individuals with PSC.^10,28^ The conflicting evidence may be attributed to differences in study designs, genetic predispositions, geographic and environmental factors, as well as statistical models employed.

 Our study has several limitations that should be acknowledged. Firstly, the retrospective cross-sectional design employed in this study introduces certain limitations such as recall bias, inability to control for all potential confounding factors, and inability to establish causality. Secondly, we did not assess several known etiologies (such as toxin exposure and infections) and unknown factors that are associated with CA 19‒9 and body weight. Thirdly, the presence of inflammatory bowel diseases as a potential risk factor of CCA in PSC patients was not assessed in our study due to lack of available data. Therefore, further large-scale prospective investigations are warranted with considering these factors to provide a more comprehensive understanding of the disease. Fourthly, the sample size, while sufficient for estimating CCA prevalence, may be underpowered for multivariable logistic regression given the low number of events (29 CCA cases), potentially leading to overfitting or sparse data bias. *Post-hoc* power calculations for the key predictors (CA 19‒9 and weight loss) indicate approximately 80% power to detect the observed odds ratios at α = 0.05, but larger prospective studies are recommended for validation. Furthermore, stepwise selection methods may inflate Type I errors and lead to unstable models; however, consistency across methods mitigates this concern. Lastly, it is important to note that as a single-center study, external validity may be limited; multicenter validation is needed, particularly for subgroups (for example, by disease duration).

## Conclusion

 Our results show that a CA 19‒9 level above 46 U/mL has a relatively good predictive power for incidental CCA in PSC patients. Accordingly, patients with a CA 19‒9 level greater than this cutoff have a five-time higher risk of incidental CCA. Furthermore, weight loss was identified as another significant risk factor for development of CCA. Future prospective studies with larger samples are recommended to evaluate the predictive power of CA 19‒9 in diverse populations, particularly when combined with other viable detection strategies and when stratifying CCA by its anatomical subtypes (intrahepatic, hilar, and distal).

## References

[1] Little R, Wine E, Kamath BM, Griffiths AM, Ricciuto A (2020). Gut microbiome in primary sclerosing cholangitis: A review. World J Gastroenterol.

[2] Cooper J, Markovinovic A, Coward S, Herauf M, Shaheen AA, Swain M (2024). Incidence and Prevalence of Primary Sclerosing Cholangitis: A Meta-analysis of Population-based Studies. Inflamm Bowel Dis.

[3] Gochanour E, Jayasekera C, Kowdley K (2020). Primary Sclerosing Cholangitis: Epidemiology, Genetics, Diagnosis, and Current Management. Clin Liver Dis (Hoboken).

[4] Boberg K, Lundin K, Schrumpf E (1994). Etiology and pathogenesis in primary sclerosing cholangitis. Scand J Gastroenterol.

[5] Prokopič M, Beuers U (2021). Management of primary sclerosing cholangitis and its complications: an algorithmic approach. Hepatol Int.

[6] Guedj N (2022). Pathology of Cholangiocarcinomas. Curr Oncol.

[7] Abbas N, Quraishi MN, Trivedi P (2022). Emerging drugs for the treatment of primary sclerosing cholangitis. Curr Opin Pharmacol.

[8] Rabiee A, Silveira MG (2021). Primary sclerosing cholangitis. Transl Gastroenterol Hepatol.

[9] Bonato G, Cristoferi L, Strazzabosco M, Fabris L (2015). Malignancies in Primary Sclerosing Cholangitis--A Continuing Threat. Dig Dis.

[10] Song J, Li Y, Bowlus CL, Yang G, Leung PSC, Gershwin ME (2020). Cholangiocarcinoma in Patients with Primary Sclerosing Cholangitis (PSC): a Comprehensive Review. Clin Rev Allergy Immunol.

[11] Bao F, Liu J, Chen H, Miao L, Xu Z, Zhang G (2022). Diagnosis Biomarkers of Cholangiocarcinoma in Human Bile: An Evidence-Based Study. Cancers (Basel).

[12] Krasnodębski M, Grąt M, Wierzchowski M, Szczęśniak M, Morawski M, Zając K (2020). Analysis of Patients With Incidental Perihilar Cholangiocarcinoma: An Old and a Persistent Burden for Liver Transplantation. Transplant Proc.

[13] Ismail MF, Hirschfield GM, Hansen B, Tafur M, Elbanna KY, Goldfinger MH (2022). Evaluation of quantitative MRCP (MRCP+) for risk stratification of primary sclerosing cholangitis: comparison with morphological MRCP, MR elastography, and biochemical risk scores. Eur Radiol.

[14] Geramizadeh B, Ghavvas R, Kazemi K, Shamsaeefar A, Nikeghbalian S, Malekhosseini SA (2015). Cholangiocarcinoma Secondary to Primary Sclerosing Cholangitis in Explanted Livers: A Single-Center Study in the South of Iran. Hepat Mon.

[15] Malik S, Dbouk N, Grant LM, et al. Primary Sclerosing Cholangitis. [Updated 2026 Apr 11]. In: StatPearls. Treasure Island (FL): StatPearls Publishing; 2026 Jan-. Available from: https://www.ncbi.nlm.nih.gov/books/NBK537181/. 30725866

[16] Søgaard KK, Erichsen R, Lund JL, Farkas DK, Sørensen HT (2014). Cholangitis and subsequent gastrointestinal cancer risk: a Danish population-based cohort study. Gut.

[17] Fevery J, Henckaerts L, Van Oirbeek R, Vermeire S, Rutgeerts P, Nevens F (2012). Malignancies and mortality in 200 patients with primary sclerosering cholangitis: a long-term single-centre study. Liver Int.

[18] Lazaridis KN, Gores GJ (2006). Primary sclerosing cholangitis and cholangiocarcinoma. Semin Liver Dis.

[19] de Valle MB, Björnsson E, Lindkvist B (2012). Mortality and cancer risk related to primary sclerosing cholangitis in a Swedish population-based cohort. Liver Int.

[20] Duggan WP, Brosnan C, Christodoulides N, Nolan N, Kambakamba P, Gallagher TK (2023). Outruling cholangiocarcinoma in patients with primary sclerosing cholangitis wait-listed for liver transplantation: A report on the Irish national experience. Surgeon.

[21] Saffioti F, Mavroeidis VK (2021). Review of incidence and outcomes of treatment of cholangiocarcinoma in patients with primary sclerosing cholangitis. World J Gastrointest Oncol.

[22] Charatcharoenwitthaya P, Enders FB, Halling KC, Lindor KD (2008). Utility of serum tumor markers, imaging, and biliary cytology for detecting cholangiocarcinoma in primary sclerosing cholangitis. Hepatology.

[23] Grimsrud MM, Folseraas T (2019). Pathogenesis, diagnosis and treatment of premalignant and malignant stages of cholangiocarcinoma in primary sclerosing cholangitis. Liver Int.

[24] Lee T, Teng TZJ, Shelat VG (2020). Carbohydrate antigen 19-9 - tumor marker: Past, present, and future. World J Gastrointest Surg.

[25] Liang B, Zhong L, He Q, Wang S, Pan Z, Wang T (2015). Diagnostic Accuracy of Serum CA19-9 in Patients with Cholangiocarcinoma: A Systematic Review and Meta-Analysis. Med Sci Monit.

[26] Kuzu UB, Ödemiş B, Suna N, Yıldız H, Parlak E, Dişibeyaz S (2016). The Detection of Cholangiocarcinoma in Primary Sclerosing Cholangitis Patients: Single Center Experience. J Gastrointest Cancer.

[27] Burak K, Angulo P, Pasha TM, Egan K, Petz J, Lindor KD (2004). Incidence and risk factors for cholangiocarcinoma in primary sclerosing cholangitis. Am J Gastroenterol.

[28] Hu C, Iyer RK, Juran BD, McCauley BM, Atkinson EJ, Eaton JE (2023). Predicting cholangiocarcinoma in primary sclerosing cholangitis: using artificial intelligence, clinical and laboratory data. BMC Gastroenterol.

